# Data quality audit of the arthroplasty clinical outcomes registry NSW

**DOI:** 10.1186/s12913-014-0512-6

**Published:** 2014-11-20

**Authors:** Kurt G Seagrave, Justine Naylor, Elizabeth Armstrong, Kwong-Ming Leong, Joseph Descallar, Ian A Harris

**Affiliations:** South Western Sydney Clinical School, Faculty of Medicine, UNSW Australia, Liverpool Hospital, Liverpool, NSW 2170 Australia; Whitlam Orthopaedic Research Centre, Ingham Institute for Applied Medical Research, 1 Campbell St, Liverpool, NSW 2170 Australia; South Western Sydney Local Health District, Elizabeth St, Liverpool, NSW Australia; Ingham Institute for Applied Medical Research, 1 Campbell St, Liverpool, NSW 2170 Australia

**Keywords:** Medical audit, Medical records, Arthroplasty, Registries

## Abstract

**Background:**

The Arthroplasty Clinical Outcomes Registry NSW (ACORN) was initiated in 2012. ACORN is a registry piloting within NSW, Australia with several participating hospitals; it aims to monitor patient-centred outcomes and post-surgical complications after total hip and knee arthroplasty. Using retrospective audit methodology, we aimed to investigate the completeness and accuracy of data in ACORN.

**Methods:**

We undertook a reabstracting audit of 100 clinical records of patients who underwent surgery in 2012/2013 (50 each from hospitals A and B). These records represented 27% (100/367) of patient entries in the ACORN registry, all of which were collected at either hospital A or hospital B. Firstly, data completeness was determined by identifying the proportion of missing data in the original data pro forma. Secondly, accuracy of the initial data extraction was determined by comparing these data to reabstracted data collated by an auditor blind to the outcomes of the initial extraction. Inaccuracies were ascertained to be a disagreement between categorical variables and for continuous data, a pre-determined window of error was established. Benchmarks for data completeness and accuracy were set at 95.0%; kappa and intraclass coefficient (ICC) calculations were also utilised to supplement this analysis. In addition, registry completeness (the percentage capture of eligible patients) was also determined as part of the data quality analysis.

**Results:**

Completeness and accuracy of submitted datasets were evaluated to be 99.0% (1259/1272) and 94.0% (2159/2296) respectively for Hospital A, and 99.3% (1589/1600) and 96.1% (2444/2542) for Hospital B. The majority of accuracy discrepancies pertained to medical history data. For Hospital A, 57.1% (28/49) of variables met the accuracy benchmark of 95%; 74.5% (38/51) of variables in Hospital B met this benchmark. Of the number of patients eligible for inclusion in the registry, 93.5% (660/706) were found to be included.

**Conclusion:**

Levels of data completeness and accuracy were found to be high in the submitted datasets for both hospitals. However, important deficits were identified in the accuracy of patient comorbidities. More specific and clear data definitions, and a more thorough examination of medical records would be possible methods to improve the accuracy of deficient areas.

**Electronic supplementary material:**

The online version of this article (doi:10.1186/s12913-014-0512-6) contains supplementary material, which is available to authorized users.

## Background

Outcomes data are increasingly being used to improve the quality of care delivered by health services. The Australian Orthopaedic Association National Joint Replacement Registry (AOANJRR) commenced in 1999. As a national registry, it functions to monitor a specific outcome time to first revision of joint arthroplasty, and the registry has been successful in this aspect [1,2].

However, the AOANJRR does not provide information on the impact of arthroplasty on patient-centred outcomes (such as pain, function and quality of life), nor does it monitor specific complications post-surgery beyond prosthesis revision and mortality. In order to fill this knowledge gap, the Whitlam Orthopaedic Research Centre initiated the Arthroplasty Clinical Outcomes Registry NSW (ACORN) in 2012. This clinical registry receives information from participating hospitals, and utilises outcome tools such as the EQ-5D-5 L [3] and the Oxford Hip and Knee scores [4], and integrates concepts borrowed from international hip and knee arthroplasty registries [5,6], other Australian outcomes registries [1], and the English patient-reported outcome measures (PROMs) movement [7]. ACORN’s focus on PROMs reflects the belief that patient post-operative health status and quality of life play a significant role in decision making and outcome assessment of joint replacement surgery.

For registry data to be used in the planning, monitoring and improvement of the quality of healthcare, detailed data of sufficient quality are required [8]. Such quality may be analysed in terms of completeness and accuracy. Completeness can be assessed by the coverage of total cases on which the registry is able to collect information, or with reference to missing data present in the registry (which will be used as the definition of data completeness in this report). Accuracy is a measure of correctness of each item of data collected [8,9].

Whilst ACORN presently has data quality measures in place, the accuracy and completeness of data submitted using a purpose-built pro forma has not yet been substantiated. For registry data to be used to inform clinical decision-making, levels of completeness and accuracy must be assessed. In this study, using retrospective audit methodology, we aimed to determine the data quality in ACORN. Here, we randomly selected and reabstracted 27% of the records included in the ACORN database. Data quality refers to the level of completeness of the pro forma data fields, and the accuracy with which information in the original source file (the medical record) is extracted and subsequently recorded on the pro forma.

A complete patient record within ACORN contains three PROM tools: EuroQoL-5D-5 L dimensions, EuroQoL-VAS (Visual Analogue Scale) score and total for Oxford Hip or Oxford Knee Score. Of the 311 entries contributed by Hospital A (over the period 01/07/12 – 30/06/13), five contained at least one missing PROM element, representing 1.6% of the total patient records in ACORN for Hospital A. In comparison, Hospital B had 71 out of 349 records with at least one missing PROM element over the period 01/09/12 – 30/06/13, representing 20.3% of the total Hospital B submissions: from this, 11.5% (76/660) of patient records in ACORN contained missing PROMs data. Thus, 88.5% of patients included in ACORN completed all three baseline PROMs assessments. This is summarised in Table 1.

**Table 1:** **PROM completion and registry completeness**

**Hospital**	**Records with Completed PROM**	**Total Records**	**Completion Rate (%)**	**No. of Relevant Cases Reported at Site**	**Registry Completeness (%)**
A	306	311	98.4	317	98.1
B	278	349	79.7	389	89.7

## Methods

### Data source and collection

ACORN is a prospective, longitudinal cohort registry open to public and private hospitals in Australia that undertake planned hip and knee arthroplasty. Participants are recruited on an opt-out basis. Currently, ACORN is piloting at several hospitals in NSW, and has utilised a rolling method of hospital recruitment. The data held in the registry at the time of this audit represents two foundation hospitals contributing to ACORN. Both hospitals A and B are classified within the NSW major hospitals peer group [10]. Ethical approval for this study was granted on 13/03/13 by the Hunter New England Human Research Ethics Committee (HNE HREC) – reference no. 12/11/21/5.02.

The data collected can be divided into four main sections:

- demographic and administrative information.
- medical (co-morbid) history.
- pre-operative health status (pain, function, quality of life).
- procedure details and recovery.

The registry data collection process for both hospitals involves the completion of standardised forms, which are forwarded to the registry. The data collection process varies slightly between the two hospitals. Once each site coordinator has submitted their data (which occurs on a routine basis), a quality and completeness check is undertaken in order to identify obvious errors or missing data points, allowing for correction prior to data entry. Following data entry, data collection forms are stored and filed for use at the six-month follow-up time point.

### Data reabstraction

50 patient records were randomly selected from each hospital (A and B) in the ACORN database using a computer-generated sequence. During the time period in which the audit applied (the first six months of data submission), 179 patient records were available in the ACORN database from hospital A, and 188 available in hospital B. Sampling rates were therefore 27.9% (50/179) and 26.6% (50/188) for hospitals A and B respectively. 50 patients were chosen from each hospital under the recommendations by Altman in the selection of a minimum of 50 patients for reliability and reproducibility studies [11]. Reabstraction was limited to the demographic, administrative, medical history, procedure and acute care details, and necessarily excluded the data that was patient-reported (for example, the EQ-5D-5 L and Oxford Scores). A researcher not directly involved with the ACORN data collection, entry or analysis performed the record randomisation process. Two random sequences of numbers were generated so that 50 patient records from each hospital were used. One record from hospital A was not considered for analysis as the surgical procedure detailed was excluded from the registry.

The reabstraction process involved the auditor accessing the relevant medical records at each hospital, completing the data pro forma, and then entering the data into an electronic spreadsheet (the audit dataset). A second reviewer entered the data originally submitted by the hospital into a second spreadsheet (which will be acknowledged as the original dataset). The re-entering of the original data eliminated transcription error by the previous data entry staff member as a source of inaccuracy between the first and second auditor. By doing this, measures of data completeness and accuracy were determined for the submitted datasets to ACORN. Neither the first or second auditor accessed the data spreadsheet of the other.

The information within the original dataset was then assessed for quality in terms of data completeness. Any missing cells were scored as missing data, and these were added and scored as a proportion to give a final value for the completeness of original data collection. All variables were assessed under this data quality check.

Evaluating the accuracy of the original dataset was performed by direct comparison of the abstracted and reabstracted data. The variables included were co-morbid conditions, previous lower limb arthroplasty, the side and type of arthroplasty, and recovery details such as intra- and post-operative complications, donor blood, discharge destination and length of stay.

A discrepancy in any categorical variable (n =44 (hospital A), n =46 (hospital B)) was noted as a disagreement. Kappa values were calculated for categorical variables in order to account for random error when analysing the agreement between the two datasets. The following convention was used to interpret the Kappa values; <0, less than chance agreement; 0.01 – 0.2, slight agreement; 0.21 – 0.4, fair agreement; 0.41 – 0.6, moderate agreement; 0.61 – 0.8, substantial agreement; 0.81 – 0.99, almost perfect agreement [12]. For scalar variables such as height and weight, a reasonable window of error, defined as an acceptable range of values for which variation would not significantly impact on data calculations, was allowed for determining discrepancies [13]. A range of ±1 year was permitted for age to allow for differences in dates between forms. Height was afforded ±0.03 m and weight ±5 kg in accordance with variation in patient reports of these variables. Length of stay was afforded ±1 day of discrepancy as a result of whether the data collector identified the day of admission as day 0 or 1. Intraclass correlation calculations (ICC) (two-way random for absolute agreement) were also determined for continuous data variables [12,14]. ICC values range from 0 to 1 with 0 suggesting no agreement; and 1 as perfect agreement. Total discrepancy levels for the datasets were then determined, as well as the individual levels of discrepancy for each variable. A finite population correction was applied to the standard errors used to calculate 95% confidence intervals for kappa and accuracy estimates, with sampling rates of 27.9% (50/179) for hospital A and 26.6% (50/188) for hospital B.

To minimise the impact of transcription errors (as opposed to actual differences in data extraction), a check system was implemented during the data analysis process, where any unusual discrepancies in the two datasets were double checked against the original and audit forms simultaneously.

In addition, registry completeness data were determined to supplement the analysis of data quality. This numerator was the total number of patient records in ACORN in a defined period, with the denominator being the total number of hip and knee replacements performed at hospitals A and B. The denominator was determined by reviewing the hospital administrative database to report the total number of primary and revision hip and knee replacements performed by each hospital for the equivalent period. The administrative databases are independently managed from the ACORN project.

## Results

### Registry completeness

It was determined that 317 hip and knee arthroplasties were performed at Hospital A for the period 01/07/12 – 30/06/13. ACORN contains data on 311 of these cases. From 01/09/12 – 30/06/13, Hospital B reported 389 hip and knee arthroplasties. ACORN holds 349 of these cases. In summary, the ACORN registry captured 98.1% (311/317) of cases from Hospital A, and 89.7% (349/389) of cases of Hospital B for the defined period. This represents 93.5% (660/706) of the total number of patients eligible for inclusion in the registry. Table 1 summarises this data.

Patient records with no missing data and no discrepancies accounted for 10.2% (5/49) of records in Hospital A, and for 8.0% (4/50) of records in Hospital B, as shown in Table 2.

**Table 2:** **Number of complete and accurate records**

**Hospital**	**No. of Records Reabstracted**	**No. of Records with Discrepancy or Missing Data**	**No. of Records without Discrepancy or Missing Data**	**Percentage of Records Error-Free (%)**
A	49	44	5	10.2
B	50	46	4	8.0

### Data completeness

Thirteen missing data entries were identified for Hospital A. This provided a final data completeness value of 99.0% (1259/1272 total sections completed over 49 patient records). For Hospital B, data completeness was 99.3% (1589/1600 total sections completed over 50 patient records) on account of 11 missing entries. Missing data were not restricted to any variables in particular, and were spread evenly throughout the dataset.

### Data accuracy

For Hospital A, 137 discrepancies were identified out of a possible 2296 matching entries (40 × 49 + 4 × 27 categorical and 5 × 49 scalar variables, minus missing data). This provided a final accuracy measurement of 94.0% (2159/2296) for Hospital A. Across the 49 variables in Hospital A, 28 variables achieved the accuracy benchmark (95%), representing 57.1% (28/49) of the total as shown in Table 3. Variables with <85% agreement (discrepancy rates above 15%) included those in the comorbidity field “hypertension”, “previous THR/TKR” and post-operative outcomes “post-operative complications”, “other” and “donor blood units”. Twenty variables achieved substantial or more (≥0.6) kappa values. Lower-range kappa values (below 0.40) were calculated for the comorbidity “other lower limb arthritis” (attributable to poor agreement), and the post-operative complications “suspected superficial wound infection” and “other” (a result of low incidence levels in the original dataset). Agreement for the variable “post-operative complications” demonstrated the lowest agreement value (73.5% - 36/49 records agreed). 71 discrepancies in the medical history data section made up 51.8% (71/137) of total discrepancies found. “See Additional file 1 – Hospital A (1) and (2)”.

**Table 3:** **Discrepancy benchmarks in hospitals A and B**

**Hospital**	**No. of Variables Reviewed**	**No. of Variables with ≥95% Accuracy**	**No. of Variables with <95% Accuracy**	**Percentage of Variables Meeting Accuracy Benchmark (%)**
A	49	28	21	57.1
B	51	38	13	74.5

In summary, 89.8% (44/49) of patient entries assessed for hospital A contained at least one data field discrepancy or missing data field. 94.0% (2159/2296) of individual data fields assessed were determined to be accurate. 57.1% (28/49) of variables assessed met the 95.0% benchmark for data accuracy.

The accuracy of data collected from Hospital B was determined to be 96.1% (2444/2542) due to 98 discrepancies identified from a possible 2542 matching entries (46 × 49 categorical +5 × 50 continuous data, minus missing data). Discrepancy rates over 15.0% were noted in the comorbidities “GIT”, “hyperlipidaemia”, “respiratory” and “lower back problems”. Low-range kappa values were noted in the comorbidities “GIT” and “hyperlipidaemia” (on account of poor agreement); the comorbidity “CNS” and post-operative complications “bladder retention” and “other” rendered low-range kappa values on account of low incidence. Of the 51 variables tested, 38 met the 95% accuracy benchmark which represents 74.5% of the total (38/51) (as shown in Table 3). The comorbidity ”hyperlipidaemia” marked the lowest agreement value across all variables (70.0% - 35/50 records agreed). The majority of discrepancies noted were present in the medical history data section (77.6% (76/98) of total discrepancies). “See Additional file 1 – Hospital B (1) and (2)”.

92.0% (42/50) of patient entries assessed in hospital B contained at least one data field discrepancy or missing data field. 96.1% (2444/2542) of individual data fields assessed were determined to be accurate. 74.5% (38/51) of variables assessed met the 95.0% benchmark for data accuracy.

Variables that met the accuracy benchmark for both hospitals included the comorbid condition “diabetes”, the surgery type and side, and the majority of post-operative complications. Discrepancy rates over 10.0% for both hospitals were noted in the co-morbid conditions “heart”, “hypertension”, “hyperlipidaemia”, “GIT” and “lower back problems”. The majority of scalar variables for both hospitals measured an ICC greater than 0.90, with only “donor blood units” for Hospital A deviating from this trend (ICC =0.580) on account of poor agreement.

## Discussion

The levels of data completeness for both hospitals were high, with forms being completely filled in the majority of cases. Missing data appeared to be random. The consistently high levels of data completeness indicate that current systems in place to address potential issues have been successful [15].

The North American Association of Central Cancer Registries (NAACRR) have determined benchmarks of ≥90% (silver certification) and ≥95% (gold certification) to be used for determining the quality of coverage of a cancer registry [16]. From this, the research team aimed to achieve a final accuracy value of 95%. Hospital A just falls short by 1.0%, whilst Hospital B exceeds this benchmark by 1.1% [17].

There are several factors most likely to have impacted on the discrepancy levels for both hospitals. It is important to note that as part of the data analysis process of determining discrepancies, it was assumed that the auditors’ account was correct when a mismatch occurred during comparison with the original dataset. However, the impact of false positives and negatives must be considered for both the auditor and original data collector, and also the limits of the retrospective study. Our results also suggest a certain difficulty in analysing some variables, which may be derived from a variety of sources, such as the patient or a clinician. These variables included patient weight and height, and their comorbid conditions. Variability in these fields was noted within the same record, which in turn required the auditor to determine which version was correct. Disorganised form arrangement in some medical records may also have contributed to the increased likelihood of discrepancies occurring during the data extrapolation process, for both the original data collection and the audited data. Hospital B was identified as having the more structured and organised medical record, allowing data extraction to be relatively straightforward in comparison to Hospital A.

This was acknowledged as one of the key factors influencing the number of variables meeting the 95% accuracy benchmark (57.1% of variables in Hospital A as compared to 74.5% in Hospital B).

Another issue with discrepancy levels were the definitions of the data: the registry took the opinion that comorbidities must be ongoing and of sufficient severity to be treated in some way. There is a grey-area when it comes to this definition, as conditions such as asthma and foot gout were commonly detailed in medical records but were often not included in the original data collection forms. This highlights the importance of having strict and unambiguous definitions of data to be collected, and continuously reviewing such definitions in line with evaluation of the submitted data. The higher discrepancy values for particular comorbid conditions indicate that data collection staff may require additional training or information regarding the comorbidity classifications.

For scalar variables, we left some flexibility for error specifically for height and weight values. This was due to the variability of their documentation within the same record. We allowed for what we believed to be a reasonable window for both of these; the ICC values indicate that the two datasets had an otherwise high level of agreement between them. Length of stay was allowed an error of one day on account of differences as to whether the date of surgery commenced on day 0 or 1. The registry defines the date of surgery as day 0, however, this definition may have been interpreted differently by clinicians responsible for the collection of data. Amendment of the way length of stay is collected, from number of days to specific dates for surgery and discharge, would allow consistent application and calculation of this data field.

As the data collection form has undergone revision, based on feedback over the implementation period of ACORN, it is expected that data collected from the earlier versions would be of less quality than that of the newer versions. Regular revisions have been implemented to address issues such as the inputting of data onto the pro forma, as well as the information to collect and how data is requested. For example, version 1 of the data collection sheet asks if the patient had ever undergone a previous TKR or THR, and also to specify the type of operation. The more current versions further require both the side (left, right or both) of operation, as well as the specific procedure undertaken. This may have contributed to the lower levels of accuracy measured in Hospital A compared to Hospital B. With implementation of more specific and clear procedures for data collection and submission, it is anticipated that newer sites contributing to ACORN will consistently submit more accurate and complete data.

The minimum data are reviewed on a defined basis to look at which data items are not collected well, need better definition or require further training in interpretation. From this audit, it is clear that the methodology in collecting co-morbid data requires review, as the majority of accuracy discrepancies were focussed in this area. Data source verification is an ongoing challenge for registries and for sites. For now, we suggest that comorbid data be obtained via the patient denoting the medication they are currently taking and its purpose. This site coordinator may then verify the patient-reported data with the specialist, general practitioner and anaesthetic reviews embedded in the medical record.

In the long term, the registry aims to extract from administrative systems and involve direct database entry with validation rules included, which should result in decreased duplication of data collection and reduced error. For this to be successful, hospital data collection processes and organisation of medical records would also require improvement.

## Conclusions

The results indicate that data collected in the first six months of operation of ACORN from two major hospitals in NSW have high levels of data completeness and accuracy. However, though relatively infrequent, important discrepancies occurred in important areas such as comorbid conditions and complications. For these data to usefully inform practice, improvements in collection of comorbid and complication data need to occur. Clinician feedback from contributing sites should be incorporated into the quality framework for the registry to improve systems and clarify definitions in order to increase levels of data completeness and accuracy in ACORN.

## Additional file

Additional file 1:
**ACORN - Variables for hospitals A and B.** Description: contains data on accuracy, kappa and incidence values of assessed variables.

## Abbreviations

ACORN: Arthroplasty Clinical Outcomes Registry NSW
AOANJRR: Australian Orthopaedic Association National Joint Replacement Registry
THR: Total hip replacement
TKR: Total knee replacement
CNS: Central nervous system
PNS: Peripheral nervous system
GIT: Gastrointestinal
NAACCR: North American Association of Central Cancer Registries
ICC: Intraclass correlation coefficient
CI: Confidence interval
CM: Comorbidity
PC: Post-operative complication
CVS: Cardiovascular system
PE: Pulmonary embolism
DVT: Deep vein thrombosis
